# Long noncoding RNAs: a missing link in osteoporosis

**DOI:** 10.1038/s41413-019-0048-9

**Published:** 2019-03-27

**Authors:** Andreia Machado Silva, Sara Reis Moura, José Henrique Teixeira, Mário Adolfo Barbosa, Susana Gomes Santos, Maria Inês Almeida

**Affiliations:** 1 i3S—Instituto de Investigação e Inovação em Saúde, University of Porto, Porto, Portugal; 2 INEB—Instituto de Engenharia Biomédica, University of Porto, Porto, Portugal; 3 ICBAS—Instituto de Ciências Biomédicas Abel Salazar, University of Porto, Porto, Portugal

**Keywords:** Bone, Osteoporosis

## Abstract

Osteoporosis is a systemic disease that results in loss of bone density and increased fracture risk, particularly in the vertebrae and the hip. This condition and associated morbidity and mortality increase with population ageing. Long noncoding (lnc) RNAs are transcripts longer than 200 nucleotides that are not translated into proteins, but play important regulatory roles in transcriptional and post-transcriptional regulation. Their contribution to disease onset and development is increasingly recognized. Herein, we present an integrative revision on the studies that implicate lncRNAs in osteoporosis and that support their potential use as therapeutic tools. Firstly, current evidence on lncRNAs involvement in cellular and molecular mechanisms linked to osteoporosis and its major complication, fragility fractures, is reviewed. We analyze evidence of their roles in osteogenesis, osteoclastogenesis, and bone fracture healing events from human and animal model studies. Secondly, the potential of lncRNAs alterations at genetic and transcriptomic level are discussed as osteoporosis risk factors and as new circulating biomarkers for diagnosis. Finally, we conclude debating the possibilities, persisting difficulties, and future prospects of using lncRNAs in the treatment of osteoporosis.

## Molecular biology: decoding the dark matter of osteoporosis

Genomic ‘dark matter’ could improve the diagnosis and prognosis of patients with osteoporosis, as well as providing new drug targets for the disease. Only 2.3% of the human genome is translated into protein; the function of the remainder had been unclear, but recent studies suggest some of it provides instructions for making regulators of protein synthesis called long non-coding RNAs (lncRNAs). In this review, Maria Ines Almeida at the University of Porto in Portugal and colleagues explore the role of lncRNAs in bone cell differentiation, metabolism and fracture healing - and describe early studies assessing their role as diagnostic and prognostic biomarkers of osteoporosis. Future advances in genome editing may also provide opportunities to redress the balance between bone production and resorption – something that becomes skewed in osteoporosis - by targeting lncRNAs.

## Introduction

Osteoporosis is a systemic and progressive skeletal disorder affecting more than 200 million people worldwide per year^1,2^. It is characterized by a decrease in bone strength (bone mineral density [BMD] and bone quality) caused by an imbalance between bone formation and bone resorption^3^, which leads to an increase in fracture risk (referred as osteoporotic fractures)^2^. It is estimated that osteoporosis causes more than 8.9 million fractures worldwide each year^1^, and the most common sites of fracture are the hip, spine, distal forearm, and proximal humerus^4^. Importantly, osteoporotic fractures are a cause of morbidity and mortality in patients and have great impact on health care systems^4^, with costs ascending to 98 billion Euros in the EU27 in 2010 ^5^. This disease is generally age-related, being more prevalent in individuals over the age of 50^6^. Considering that global life expectancy is increasing, it is estimated that the worldwide incidence of hip fractures will increase by 3.5 times between 1990 and 2050, accounting for a total of 6.26 million fractures in 2050^7^.

In osteoporotic patients, the natural process of bone remodeling, that occurs throughout life, becomes unbalanced^2^. At the cellular level, osteoporosis is translated by an enhancement of osteoclasts activity (bone-resorbing cells), which is not counterbalanced by an increase in cellular differentiation and activity of osteoblasts (bone-forming cells)^2^. At the molecular level, deregulation of osteoprotegerin/tumor necrosis factor (TNF) receptor superfamily member 11a (TNFRSF11A/RANK)/RANK Ligand (RANKL), WNT, and bone morphogenetic protein (BMP) signaling pathways provides the basis for osteoporosis and bone fragility onset^2^.

Several causes for osteoporosis have been identified, including hormone deficiency, genetic disorders, use of certain medication regimens, age, immobilization, diseases such as rheumatoid arthritis, frequent smoking, elevated alcohol consumption, and dietary deficiencies in vitamin D and calcium^2,8^. These causes are also risk factors for the occurrence of fragility fractures, along with ethnic background, low BMD, low-body weight, hyperkyphosis, falling, and history of previous fractures (osteoporotic or not)^8^. Fracture risk assessment can be stratified using FRAX system that integrates distinct clinical factors and can be used with or without BMD evaluation^3,4^. Current options for osteoporosis management aim to prevent bone fractures^9^, and are mainly based in drug agents, most commonly bisphosphonates (Alendronate, Risedronate, Zoledronic acid, and Ibandronate)^3^, which are antiresorptive drugs that inhibit osteoclast function^2,9^. Although bisphosphonates are estimated to reduce fractures by 40%–70%, several limitations have been reported, including acute renal failure, gastrointestinal intolerability, musculoskeletal pain and, in rare cases, an increased risk of fracture upon their long-term use, particularly of atypical femoral fractures and osteonecrosis of the jaw^3,9^. Instead of limiting osteoclasts function, other pharmacological therapies aim to stimulate bone formation using anabolic agents, particularly parathyroid hormone treatment, for osteoporosis cases with severe risk of fracture^10^. For postmenopausal women, which are considered a risk group due to bone loss acceleration after menopause^4^, hormone replacement (estrogen and progestin therapy, estrogen therapy alone or selective estrogen receptor modulators) is still a first line clinical choice of treatment^2,3^. However, effects such as increased breast cancer risk^11^ have been reported, though literature is still controversial regarding this topic^12^. Intake of calcium and vitamin D is often used for osteoporosis prevention, but it is not fully effective in avoiding the development of this condition^2^.

**Fig. 1:**
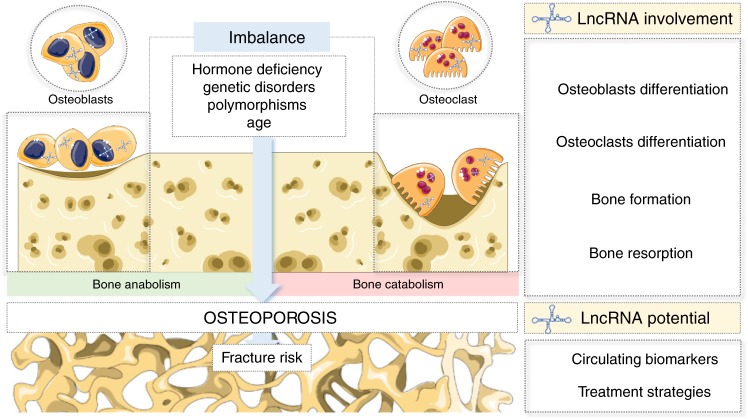
Long noncoding RNA are crucial mediators of the bone remodeling process, which is disrupted in osteoporosis

Therefore, understanding the etiology and molecular mechanisms of bone damage in osteoporosis might help to find more effective treatments to prevent microarchitectural deterioration of bone tissue and maintain bone homeostasis. Long noncoding RNAs (lncRNAs) have emerged as new key regulatory molecules, whose expression is deregulated in disease, and in the next sections we will discuss why their study should be pursued in the case of osteoporosis (Fig. 1). Firstly, the molecular regulation exerted by lncRNAs in bone-forming and bone-resorbing cells will be extensively detailed, and their involvement in biological events occurring as consequence of osteoporosis, as it is the case of fragility fractures, will be broadly discussed. Furthermore, the different pre-clinical animal models of osteoporosis currently available to study the involvement of lncRNAs in this disease are also described. Secondly, the value of lncRNAs as biomarkers for osteoporosis diagnosis/prognosis will be critically debated, a recent question that only few studies have explored so far. Moreover, the contribution of lncRNA-single nucleotide polymorphisms (SNPs) to osteoporosis and fractures will also be addressed. Finally, we explain how lncRNA modulation may be achieved, discussing the advantages and drawbacks of each approach and proposing new delivery strategies that may be tested in future clinical trials, in order to yield more efficient therapies for osteoporosis.

### **The vast and heterogeneous class of lncRNAs: definition, classification and functions**

Recent developments in genomic analysis technologies revealed that about 85% of the human genome is transcribed^13,14^, but only approximately 2.3% of the human genome accounts for messenger RNA (mRNA) and translate into proteins^15^. Thus, the large majority of human transcripts does not encode for proteins^13,14^. Still, while human protein-coding genes have been extensively explored over the last decades, the function of noncoding RNA (ncRNA) only recently has started to be dissected^16–18^. NcRNA research witnessed a remarkable progress from the findings that microRNAs (miRNAs)—a class of “small ncRNA” with approximately 20 nt length—function as negative post-transcription regulators of gene expression and have a direct impact on human diseases^19^. Presently, thousands of studies have been published showing the importance of miRNAs as diagnostic, prognostic, and therapeutic tools and significant efforts have been made to translate these findings into the clinics^19^. Besides miRNAs, the class of “small ncRNA”, includes transcripts categorized as small nuclear RNA, small nucleolar RNA, piwi-interacting RNA, and small interfering RNA (siRNA). However, the most predominant and heterogeneous class of ncRNAs transcripts is by far “long ncRNAs”^20,21^.

The most commonly used definition of a lncRNA is an RNA transcript longer than 200 nt that does not translate into a protein^20^. lncRNAs can be up to several thousand base pairs in length. However, standardization of lncRNAs nomenclature and classification has not been an easy task^20^, especially considering that the threshold of 200 nt was set based only on a technical convenience (RNA isolation protocols using silica columns) rather than a biological reason^20,22^. In addition, the classical definition of nonprotein-coding genes as sequences with open reading frames (ORF) less than 100 amino acids is also far from ideal as small ORF can synthetize small peptides^20^, and long transcripts with known noncoding functions might contain potential ORFs^23^. Spizzo et al. propose lncRNA class to include all noncoding transcripts that do not fit into “small ncRNA” class or into “structure ncRNA” class, such as transfer RNA and ribosomal RNA^20^. The definition and classification of lncRNAs is extensively detailed by Laurent et al.^24^ and Spizzo et al.^20^.

One commonly used subclassification for lncRNAs is their genomic location and position in relation to protein-coding genes. lncRNAs are considered intergenic when located in “gene deserts” that do not lie within or overlap with protein-coding gene loci, such as lincRNAs—long intergenic (also called intervening) ncRNAs—e.g., X-inactive specific transcript (XIST), MALAT1, NEAT1, and MIAT23. Other lncRNAs span within the same regions as protein-coding genes. In this case, lncRNAs can be classified according to their localization with respect to the known protein transcripts as (1) intronic, when located in the same region of protein-coding genes introns, (2) exonic, when covering protein-coding exons, or (3) overlapping, when the protein-coding transcript lies within an intron of the lncRNA^25^. lncRNAs can additionally be grouped as antisense (opposite orientation of coding genes) or sense RNAs (same orientation as coding genes), or bidirectional^20^.

LncRNAs share many common features with coding transcripts. Regarding subcellular localization, lncRNAs can be nuclear, cytoplasmatic, or equally present in both compartments^25^. Moreover, lncRNA are frequently (but not always) polyadenylated and normally transcribed by RNA polymerase II^20,25^. Similarly to coding transcripts, lncRNA have epigenetic markers^26^ and may contain polymorphisms^27^.

Presently, NONCODEv5 (a comprehensive database of ncRNAs, especially lncRNAs) accounts for 548 640 lncRNA in 17 different species, including additional 21 304 entries in the last 2 years^28^. Considering that next-generation sequencing techniques are now commonly used to detect lncRNA transcripts in a variety of animal^28^ and plant species^29,30^, which is partially driven by the reduction of costs associated to these methodologies^28^, it might be expected lncRNA data annotation to further expand in the following years. Thus, understanding lncRNA functions is now central to rapidly advance this research field.

Currently, lncRNAs are known to act as chromatin, transcriptional, and post-transcriptional regulators^31^. Regarding chromatin remodeling, lncRNA are capable to control chromatin structure by directly interacting with chromatin-modifying enzymes and nucleosome-remodeling factors, and to recruit chromatin-remodeling complexes to specific chromatin loci and mediate epigenetic modification^32,33^. For instance, lncRNA HOTAIR plays a vital role in chromatin regulation, since it recruits and has the ability to bind to both Polycomb repressive complex 2 (PRC 2) and lysine-specific histone demethylase 1A (LSD1), and coordinates their targeting to histone H3K4-demethylation and H3K27-trimethylation, which affect chromosome condensation and therefore gene silencing^34^. In addition, lncRNA XIST and Air can recruit chromatin-remodeling proteins, such as PRC 2 and complex G9a, respectively, and induce the silencing of specific genes, by turning them inaccessible to the transcription machinery^35,36^.  Regarding the regulation of the transcriptional process by lncRNAs, the mechanisms are diverse. For instance, lncRNAs can act as transcriptional regulation factors by recruiting transcription factors, as it is the case of lncRNA Evf2 that recruits transcription factor Dlx2, forming a complex and inducing in this way the expression of the Dlx5 and Dlx6 homeobox genes^37^. LncRNAs can also interact directly with some basic components of the RNA polymerase II machinery, controlling their binding and/or repression capacity, depending on the type of interaction^38,39^. Moreover, enhancer-associated lncRNAs are able to modulate gene expression both in *cis* and *trans*^31^. Regarding the ability of lncRNAs to post-transcriptionally regulate mRNAs, this process partially results from their capacity of hybridization with complementary sequences. Additionally, lncRNAs can function as sponges of miRNAs. Specifically, lncRNAs may contain miRNA recognition elements and sequester miRNA due to sequencing complementarity, avoiding miRNA to target mRNA, which in turn may cause an increase in the expression of the coding transcripts targeted by those miRNAs^21,40^. These lncRNAs are known as “competing endogenous RNAs” because they can compete with the miRNA targets^40^. On the other hand, it should be noted that 50% of miRNAs are produced from lncRNA transcripts. Moreover, lncRNAs can directly target mRNAs for degradation and are implicated in post-transcriptional regulatory steps such as pre-mRNA splicing, mRNA capping, polyadenylation, and regulation the nuclear trafficking^22,41^.

One of the reasons that can justify the delay on assessing lncRNAs functionality is related to their poor sequence conservation, compared with coding genes or miRNAs. Nonetheless, hundreds of segments larger than 200 nt have been identified by Bejerano et al. as 100% conserved between orthologous regions of human, rat, and mouse genomes^42^, and some are located in regions that do not encode for proteins. Importantly, these transcripts (“transcribed ultra-conserved regions”) are altered in disease, particularly in leukemia and carcinomas^43^. Nevertheless, for a large portion of lncRNAs there is no sequence conservation between species or conservation is restricted to short-sequence stretches^44,45^. Still, the lack of conservation does not suggest a lack of function^44,45^. Besides base-pair sequence, the structure, function, and expression from syntenic loci should be considered when analyzing lncRNA conservation^44^.

In humans and in other species, lncRNA dysregulation impacts key cellular functions. This include mechanisms such as apoptosis^46^, cell proliferation^47,48^, angiogenesis^49^, cell migration^47,50^, and cell differentiation^51^, among others. The recognition of their involvement in pathogenesis turned lncRNAs into potential therapeutic targets. LncRNAs have been described to play essential roles in various human diseases, including cancer (e.g., breast^52^, liver^53^, prostate cancer^54^, and leukemia^43^), cardiac^55^, or neurodegenerative diseases^56^. However, the involvement of lncRNAs in osteoporosis only recently started to be revealed. In the following sections, we will address the role of lncRNAs in different cell types and mechanisms relevant to osteoporosis.

### Regulatory roles of lncRNAs in osteogenic differentiation

Whole transcriptome profiling studies revealed that lncRNAs are highly implicated in the osteogenic differentiation process. The decreased capacity of mesenchymal stem/stromal cells (MSC) to commit toward and differentiate into the osteogenic lineage contributes to the insufficient bone formation observed in osteoporosis^57,58^. Wang et al. identified 1 206 differentially expressed lncRNAs (at least twofold) in human bone-marrow (BM)-derived MSC after 14 days of in vitro osteogenic differentiation compared with undifferentiated MSC^59^. Considering lncRNA categorization according to transcripts location, 106 were classified as sense, 162 as antisense, 111 as intronic, 54 as bidirectional, and 253 as intergenic. Bioinformatic tools identified 48 differently expressed lncRNAs with potential enhancer-like functions^59^. Two candidate lncRNAs, namely H19 and uc022axw.1, have been validated as up-regulated throughout the differentiation process^59^. Another study using microarray data on BM-derived MSC showed a total of 1 408 differently expressed lncRNAs at day 7 of osteogenic differentiation compared with non-stimulated MSC, specifically 785 upregulated, and 623 downregulated lncRNA transcripts^60^. Among those, lncRNA XR-111050 is of particular interest since it is able to enhance osteogenic differentiation of MSC through up-regulation of osteogenic markers, such as Collagen type I alpha 2 chain (COL1A2), bone gamma-carboxyglutamate protein/osteocalcin (BGLAP/OCN), osteopontin (OPN/SPP1) and Runt-related transcription factor 2 (RUNX2). On the contrary, XR_111050 silencing results in a decrease of mineralization and calcium quantification in vitro^60^. Furthermore, Qiu et al.^61^ found 433 and 232 lncRNAs continuously upregulated and downregulated, respectively, during 21 days of human BM-derived MSC osteogenic differentiation process. Finally, analysis of mouse pre-osteoblast differentiation RNA-sequencing data revealed lncRNA expression is timely controlled and presents distinct lncRNA patterns between early and late stages of differentiation^62^.

Although lncRNA whole transcriptome analysis is important to understand the extent to which lncRNAs are implicated in osteogenic differentiation, exploring the role of specific candidates is essential to refine lncRNAs relevant for potential clinical use.

### lncRNAs as osteogenic differentiation inhibitors

**Fig. 2:**
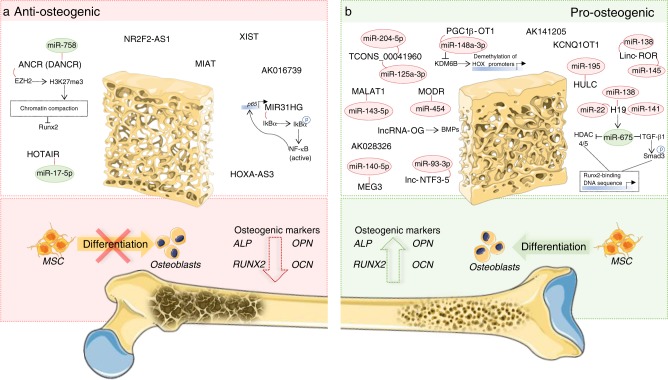
Long noncoding RNAs act as inhibitors (**a**) or promoters (**b**) of osteogenic differentiation process. lncRNAs are key transcriptional and translational regulators that may act mainly as modulators of chromatin architecture, as ligands to activators/repressors of gene promoters, as source transcripts to other regulatory RNAs, and as competing endogenous RNAs to pro-osteogenic microRNAs (green circles) and anti-osteogenic microRNAs (red circles), controlling the expression of protein-coding genes implicated in osteogenic differentiation by different mechanisms

Several lncRNAs have been shown to inhibit the process of osteogenesis (Fig. 2). One of the first studies addressing the function of specific lncRNAs in osteogenic differentiation found the anti-osteogenic role of lncRNA ANCR (alias DANCR)^63^. Using a human fetal osteoblastic cell line hFOB1.19, authors showed that ANCR downregulation induces the expression of pro-osteogenic genes, including ALP, OCN, and RUNX2^63^. Mechanistically, ANCR physically interacts with “Enhancer of zeste 2 polycomb repressive complex 2 subunit” (EZH2), which catalyzes histone methylation H3K27me3, repressing RUNX2 gene expression^63^. Therefore, ANCR indirectly plays a role as a chromatin regulator. Later, Jia et al.^64^ demonstrate the anti-osteogenic function of ANCR in periodontal ligament stem cells and showed that downregulation of ANCR activates the canonical WNT signaling pathway, which induces RUNX2 expression. Peng et al.^65^ further proposes that ANCR could act as a sponge for miR-758, a pro-osteogenic miRNA. In dental tissue-derived stem cells, ANCR inhibition promoted osteogenesis but also adipogenesis and neurogenic differentiation, which raises concerns about the effect of ANCR on lineage commitment^66^. In addition, ANCR knockdown could enhance osteogenic marker genes in a human bone-marrow stromal cell line^67^. This process is mediated by p38 MAPK pathway, since ANCR overexpression resulted in a decrease of p38 phosphorylated form^67^. Presently, the effect of ANCR knockdown in bone formation in vivo remains to be determined.

Another lncRNA identified as anti-osteogenic is HOTAIR. Expression levels of this transcript are reduced in BMP-2-induced osteogenic differentiation^68^. While silencing of HOTAIR increases RUNX2 and COL1A1 expression, its overexpression reduces mRNA levels of these genes^68^. This effect is mediated by miR-17-5p, a pro-osteogenic miRNA^69^, and by its downstream target SMAD family member (SMAD) 7^68^. Specifically, downregulation of HOTAIR contributes to the decrease of DNA methylation levels in miR-17-5p promoter, which consequently causes miR-17-5p upregulation. Authors also show that HOTAIR-expression levels were increased in BM samples isolated from patients with nontraumatic necrosis of femoral head compared with patients with osteoarthritis and healthy donors^68^, which further sustains the potential of HOTAIR as a therapeutic target in bone-related diseases with impaired osteogenic differentiation. Other authors have also shown that HOTAIR silencing increased osteogenic differentiation in human derived MSC^70^. It is known that HOTAIR plays critical roles in gene regulation and chromatin dynamics, via interaction with histone methylase (PRC2) and histone demethylase (LSD1)^71^, so other mechanisms of action on the context of osteogenic differentiation are a topic of interest for future investigation.

Another negative regulator of osteogenic differentiation is lncRNA ENST00000502125.2 (NR2F2-AS1). Its downregulation causes an increase in ALP staining and Alizarin Red S staining, while its overexpression causes the reversed effects^61^.

In a recent study, XIST was also identified as an anti-osteogenic lncRNA, with its expression being decreased in rat BM-MSC at least in the first 7 days of osteogenic differentiation induction. Furthermore, the authors of this study demonstrated that XIST overexpression impairs the expression of osteogenesis markers at the gene and protein level, and reduces MSC ALP activity and mineralization in vitro. The opposite effects were observed upon XIST knockdown^72^.

Few studies have addressed the impact of anti-osteogenic lncRNAs in vivo. Jian et al. showed that knockdown of lncRNA MIR31HG (a lncRNA transcribed by the same promoter as miR-31^73^) in human adipose derived stem cells (hASC) promoted bone formation in vivo upon cell subcutaneous implantation^74^. These authors studied the impact of the lncRNA on bone differentiation in an inflammatory environment and showed that MIR31HG expression delayed osteogenic differentiation of hASCs, whereas its knockdown significantly promoted the osteogenesis in hASC^74^, classifying this lncRNA as anti-osteogenic. Specifically, MIR31HG is upregulated by inflammatory cytokines via NF-kB, through p65 subunit that binds to MIR31HG promoter. On the other hand, MIR31HG physically binds to IkBα (an NF-kB inhibitor) and participates in its phosphorylation, causing NF-kB activation, in a positive-feedback loop between MIR31HG and NF-kB. Therefore, MIR31HG is a good target candidate to enhance bone formation in bone tissue engineering strategies. The lncRNA MIAT was also suggested to be an inhibitor of hASC osteogenesis in an in vivo model of heterotopic bone formation. Upon induction of hASC osteogenic differentiation in vitro, the expression levels of MIAT decrease along time of differentiation and MIAT knockdown in hASC via short-hairpin RNA (shRNA) increases mineralization and the expression of different osteogenic differentiation protein markers. Accordingly, the subcutaneous implantation of a collagen scaffold doped with hASC transduced with shRNA targeting MIAT, into the back of mice, resulted in increased new bone formation compared with mock-transduced hASC^75^. In another study, in vivo inhibition of AK016739 via siRNA rescued calvarial bone formation in an osteoporosis model of ovariectomized mice, revealing this lncRNA has an anti-osteogenic role^76^. Regarding MSC lineage commitment, lncRNA HOXA-AS3 was shown to be a regulator of adipogenesis and osteogenesis processes, acting as an anti-osteogenic lncRNA. In vitro, HOXA-AS3 expression promotes adipogenesis while it inhibits osteogenesis of MSCs. This process is mediated by EZH2 that binds to HOXA-AS3 and interferes with RUNX2 gene repression. Specifically, silencing of HOXA-AS3 leads to the reduction of EZH2 binding to the promoter region of RUNX2 gene, and to the decrease of H3K27me3 levels, which induces RUNX2 expression^77^. In agreement, in vivo data shows that depletion of HOXA-AS3 promotes hMSCs-mediated heterotopic bone formation^77^ .

### lncRNAs as osteogenic differentiation promoters

LncRNAs can be inducers and positive mediators of osteogenesis (Fig. 2). This is the case of lncRNA AK141205, which levels are positively regulated by the osteogenic growth peptide (OGP), an osteogenic differentiation promoter, in mouse-derived MSC^78^. The increase in ALP activity, the number of calcium salt nodules, and the expression levels of RUNX2, OPN, and OCN caused by OGP can be reversed by AK141205 knockdown, suggesting it promotes osteogenesis^78^. AK141205 is also able to induce CXCL13^78^, a pro-osteogenic chemokine^79^, by increasing H4 histone acetylation and by suppressing histone deacetylase (HDAC) 1^78^. Importantly, the effects of AK141205 upon OGP stimulation can be reversed by CXCL13 silencing, revealing the involvement of AK141205/CXCL13 axis in osteogenic differentiation^78^. High-glucose levels that impair osteogenic differentiation, which is a critical condition in patients suffering from hyperglycemia^80^, can decrease AK028326/CXCL13 expression axis in a time-dependent manner^81^. Increased expression of AK028326 was able to revert the negative effects of high glucose in osteogenic differentiation by inducing expression of osteogenic markers such as RUNX2, OPN, and OCN, and by increasing ALP activity and mineralization^81^. These effects were abrogated by CXCL13 silencing, which shows that CXCL13 is necessary to support the pro-osteogenic role of AK028326^81^.

Moreover, MEG3—a paternally imprinted gene^82^—acts as a pro-osteogenic lncRNA. Its role in MSC biology was initially identified in a study analyzing MSC derived from patients suffering from multiple myeloma, which have reduced MEG3 levels compared with normal donors^83^. While exploring its function, Zhuang et al.^83^ found that MEG3 knockdown inhibits osteogenic differentiation through the reduction of the markers RUNX2, Sp7 transcription factor/Osterix (Sp7/Osx) and OCN at the transcription level, and the decrease in the number of mineralized nodules, while its upregulation by a lentiviral system caused the opposite effects. Most interesting, these effects are caused through the regulation of the transcriptional activity of BMP4 that is implicated in osteoblast maturation^83,84^. Specifically, MEG3 expression disrupts the interaction between BMP4 promoter region and its negative regulator SOX2, causing BMP4 direct activation. As a consequence, both BMP4 transcription levels and secreted protein levels are increased upon MEG3 overexpression^83^. An independent study confirmed the involvement of MEG3 in the osteogenic lineage^85^. Specifically, authors show that its knockdown in hASC promotes adipocyte differentiation, while it inhibits osteogenic differentiation, as assessed by ALP and Alizarin Red S staining, and by analysis of RUNX2 and OCN^85^. This effect may be mediated by miR-140-5p, an anti-osteogenic miRNA, which expression inversely correlates with MEG3 levels^85^.

H19 has also been shown to act as a pro-osteogenic gene. Independent studies demonstrated that H19 is upregulated during osteogenic differentiation of human MSC^83–85^, and it promotes bone formation in vivo^86,87^. Scaffolds^86^ and resorbable bone graft substitute^87^ loaded with H19-overexpressing MSC were able to enhance ectopic bone formation in mice^86,87^. Considering H19 encodes the primary transcript of miR-675, Huang et al.^86^ proposed that both H19 and miR-675 were upregulated during the differentiation process and could downregulate transforming growth factor beta 1 (TGF-β1), an inhibitor of osteoblast full differentiation, via HDAC4/5 and p-SMAD3 that are knocked-down by H19/miR-675 overexpression. Interestingly, miR-675 directly targets TGF-β1 in the 5′ untranslated regions and in coding regions^86^. Previous studies have shown that TGF-β1 activates SMAD3 through phosphorylation, which then recruits HDAC4/5, which have HDAC activity, and forms complexes to inhibit osteogenic differentiation gene expression^88^, such us RUNX2 and osteocalcin. In addition, miR-675 was also shown to downregulate HDAC4/5 expression^86^. Taken together, these results point to a transregulatory role of H19 in osteogenic differentiation^86^. In contrast, a study by Liang et al.^87^ proposes overexpression of miR-675-5p to suppress osteogenic differentiation and miR-675-5p to negatively regulate H19 through direct binding in a feedback loop mechanism. Moreover, H19 acts as a ceRNA^87^ and a sponge for miR-141 and miR-22, both negative regulators of osteogenic differentiation. H19 could increase the expression of β-catenin, which is a miR-141 and miR-22 direct target, and activate Wnt/β-catenin pathway^87^. Finally, tension-induced osteogenic differentiation of MSC was also able to upregulate H19. Enhancement of ALP, RUNX2, OPN, and OCN expression induced by mechanical tension is abrogated by H19 knockdown^89^. H19 has binding sites for miR-138, and thus it also functions as a ceRNA for this miRNA. H19 prevents miR-138 from targeting protein tyrosine kinase 2 (PTK2) and, consequently, impairs the protein levels of focal adhesion kinase FAK, a key molecule in the mechanotransduction pathway for osteogenic differentiation that is encoded by PTK2^89^. Analysis of other potential miRNA biding sites for H19 could unravel additional pathways regulated by this lncRNA.

In a study by Tang et al.^90^, lncRNA-OG was newly identified as a pro-osteogenic lncRNA. Its expression was shown to gradually increase during osteogenic differentiation of human BM-MSC in vitro, at least during the first 10 days of differentiation induction. In accordance to these results, BM-MSC overexpressing lncRNA-OG greatly promoted in vivo ossification in a mouse model of subcutaneous heterotopic bone formation, promoting the formation of functional osteoid. Moreover, downregulation of lncRNA-OG in vitro decreased gene expression of ALP, RUNX2, OSX, and OCN, inhibiting also ALP activity and mineralization. This pro-osteogenic effect of lncRNA-OG was attributed to its capacity of promoting the expression of several proteins of the BMP family^90^.

A pro-osteogenic role was also demonstrated for the lncRNA TUG1. In the work of He and colleagues, TUG1 expression was increased upon induction of osteogenic differentiation of human periodontal ligament mesenchymal stem cells. However, the simultaneous knockdown of TUG1 upon treatment with pro-osteogenic stimuli impaired cell capacity to differentiate into the osteogenic lineage, in a process dependent of the RNA-binding protein Lin28A^91^.

Several additional lncRNAs exert a pro-osteogenic function by acting as miRNA sponges. This is the case of TCONS_00041960^92^. Its overexpression increased osteogenic-specific markers, while decreasing adipocyte-specific markers, by competing with the osteogenesis promoter RUNX2 and with the adipogenesis inhibitor GILZ for the interaction with miR-204-5p and miR-125a-3p, respectively^92^. Importantly, authors showed that TCONS_00041960 expression was down-regulated in rat BM-derived MSC upon treatment with a glucocorticoid^92^. Considering that continued intake of glucocorticoids is a known risk factor for osteoporosis and bone fracture^93^, TCONS_00041960 can be a relevant clinical target. Recently, PGC1β-OT1 was also identified as regulator of MSC lineage specification via miRNA sequestration. Downregulation of PGC1β-OT1 in vitro and in vivo promoted adipogenic differentiation of mouse cells, while inhibiting osteogenic differentiation, confirming this lncRNA as pro-osteogenic^94^. This effect was mediated by PGC1β-OT1 binding to miR-148a-3p, impairing its repression of KDM6B, a histone demethylase described to participate in the demethylation of HOX genes promoters, being thus a positive regulator of osteogenesis by indirectly controlling chromatin architecture^94^. A miRNA-sponge function was also described for lncRNA linc-ROR, a pro-osteogenic transcript in human BM-MSC that directly targets miR-138 and miR-145, both suppressors of the Wnt/β-catenin signaling and negative regulators of osteogenic differentiation^95^. Also, MALAT1 is a positive regulator of human MSC osteogenic differentiation through miRNA binding. In this case, overexpression of MALAT1 induces the increase in OSX expression, which can be abrogated by miR-143 expression. This process is mediated by MALAT1-miR-143 direct binding^96^. In rat BM-MSC, the lncRNA HULC promotes osteogenic differentiation by enhancing the activation of Wnt/β-catenin and p38MAPK pathway through the downregulation of miR-195^97^, a known anti-osteogenic miRNA^98^. HIF1α-AS2 was also demonstrated to promote osteogenic differentiation of human ASC by conditioning miRNA activity. Overexpression of this lncRNA abolishes the inhibitory action of miR-665 upon IL-6, which in turn is a promoter of osteogenic differentiation of hASC by activating the PI3K/Akt signaling pathway^99^. Interestingly, an earlier work proposed HIF1α-AS1, encoded in a genomic vicinity of HIF1α-AS2, as a promoter of human BM-MSC osteogenic differentiation via upregulation of HOXD10, and as a result of sirtuin-1 inhibition^100^. Although this study did not actually demonstrate the effect of HIF1α-AS1 in promoting MSC osteogenic differentiation, these two works suggest that HIF1α-AS1 and HIF1α-AS2 may have a concerted regulatory action upon osteogenesis. On the other hand, Chen et al.^101^ demonstrated that HIF1α-AS2 is an inhibitor of osteogenic differentiation of human periodontal ligament cells under hypoxia conditions. Therefore, it is clear the necessity for future studies to further unravel the regulatory network of HIF1α-AS1 and HIF1α-AS2 on osteogenesis in different conditions. MODR and lnc-NTF3-5 are also described as promoters of osteogenic differentiation in human maxillary sinus membrane stem cells, acting as a sponge for miR-454 and miR-93-3p, respectively, both of which are able to target RUNX2^102,103^. Further studies are still needed to confirm a similar effect of these lncRNAs in conventional MSCs.

Finally, lncRNAs were found to act as mediators of effects of compounds with bone protective properties, such as resveratrol^104^. In mouse MSC with compromised osteogenic differentiation capacity caused by polymethylmethacrylate (PMMA) particles, resveratrol alleviated PMMA-mediated osteogenic inhibition, through positive regulation of lncRNA KCNQ1OT1. In vitro studies revealed that KCNQ1OT1 could promote osteoblastic differentiation even in presence of anti-osteogenesis PMMA particles^104^ and upregulate β-catenin expression through specific interaction between KCNQ1OT1 and β-catenin protein^104^.

### Regulatory roles of lncRNAs in osteoclastogenesis

Osteoclasts are cells originated from hematopoietic stem cells through the myeloid lineage, sharing the same precursors as monocytes/macrophages, and which are responsible for bone resorption^105^. The physiological differentiation of a common myeloid precursor into the osteoclast or macrophage lineage is divergent, with osteoclast differentiation and survival being mediated by macrophage-colony stimulating factor (M-CSF) and by receptor activator of NF-κB ligand (RANKL), which have distinct roles. On one hand, M-CSF is essential for commitment of hematopoietic stem cells in the osteoclast lineage, proliferation of precursors, and osteoclast survival^105^. On the other hand, RANKL binds to RANK receptor leading to the recruitment of TNF receptor-associated factor 6 (TRAF6), and consequent activation of pathways and molecules (e.g., NF-κB, MAPKs, PI3K/AKT, AP-1 transcription factor family, and NFATc1) that promote expression of pro-osteoclastic genes, allowing the fusion of precursors and the maturation of multinucleated osteoclasts^105^. Osteoblasts and osteoclasts communicate via different mediators and mechanisms, and their crosstalk and concerted action are essential for bone health and recovery upon injury. For instance, pre-osteoblasts produce mediators, like RANKL, promoting osteoclast differentiation, while osteoclasts release factors that are incorporated in the bone matrix, such as TGF-β1 and BMPs, enhancing osteogenic differentiation^106^. Although deregulation of osteoclasts differentiation and activation is a hallmark of osteoporosis, few studies so far explored the role of lncRNAs in osteoclastogenesis.

The first report addressing the functions of lncRNA in osteoclastogenesis evaluated the differences in the profile of monocyte/macrophage mouse RAW264.7 cell line in distinct stages of osteoclast differentiation/maturation, namely monocytes to pre-osteoclasts (TRAP-positive mononucleated cells); pre-osteoclasts to mature osteoclasts (low number of multinucleated cells, increased cell fusion and bone resorption activity); and activation of mature osteoclasts (multinucleated cells, highest membrane merge rate, and most efficient bone resorption activity)^107^. Approximately, the same number of lncRNAs has been identified as differently expressed between the different stages of osteoclastogenesis compared with undifferentiated cells, namely 4348, 4602, and 5840 lncRNAs, in pre-osteoclasts, mature osteoclasts, and activated osteoclasts, respectively^107^. Further analysis revealed that 170 lncRNAs were significantly upregulated, while 348 lncRNAs were significantly downregulated in at least twofold in all stages of osteoclastogenesis^107^. These results show that lncRNA expression profile is highly regulated during osteoclastogenesis. Authors also found that two downregulated lncRNAs, Gm12310, and Gm12308 are associated with tumor necrosis factor ligand superfamily member (Tnfsf) 12 and Tnfsf13 protein-coding transcripts, which have previously been implicated in osteoclastogenesis^107,108^. A second study following the microarray results, explored the involvement of lncRNA AK077216 in osteoclastogenesis^109^. This lncRNA is significantly upregulated during osteoclastogenesis and in bone marrow and spleen tissues of OVX mice. In vitro, it promotes osteoclast differentiation and enhances osteoclast bone resorption of RAW264.7 cells^109^. Importantly, lncRNA AK077216 upregulates NFATc1^110^, a master regulator of RANKL-induced osteoclast differentiation, and this effect is mediated by NIP45, which is suppressed by AK077216. Furthermore, authors also show that c-Fos, a key molecule in osteoclast-macrophage lineage determination, is increased at both mRNA and protein level, in AK077216-overexpressing cells^109^. Considering mice with c-Fos absence are devoid of multinucleated osteoclasts, but have increased number of bone-marrow macrophages^111^, future studies could address a potential impact of lncRNA AK077216 on osteoclast-macrophage lineage specification.

Also using RAW264.7 cells as a model, Lee et al. explored the lncRNA regulatory function in monosodium urate monohydrate (MSU)-induced osteoclast differentiation, when cells were concomitantly stimulated with RANKL and M-CSF. The presence of MSU crystals in the presence of RANKL has been previously described to increase osteoclast differentiation^112^, compared with the presence of RANKL alone. Authors found several osteoclasts lineage-specific lncRNAs enhanced by MSU were co-expressed with their neighboring protein-coding genes. Particularly, lncRNA-Jak3 was found to be up-regulated at three stages of osteoclast differentiation, namely pre-osteoclasts, mature osteoclasts, and activated osteoclasts. In vitro, inhibition of lncRNA-Jak3-induced downregulation of Jak3, Nfatc1, and Ctsk osteoclasts-related genes. Thus, lncRNA-Jak3 may be a potential target candidate for MSU-induced osteoclast activation, and its role in physiological osteoclast differentiation should also be further investigated. Recently, lncRNA AK131850 was also described to be involved in the different stages of osteoclastogenesis^113^. Surprisingly, this lncRNA is a natural antisense transcript of VEGF and it can modulate endothelial progenitor cells^113^. AK131850 acts as an endogenous sponge for miR-93-5p, which alleviates the repression on VEGF expression and, consequently, promotes proliferation, differentiation, migration, and tube formation of endothelial progenitor cells^113^. Future studies should be performed aiming to validate lncRNA candidates during osteoclastogenesis in human primary samples. This is of crucial importance considering the lack of conservation among human and mouse for the majority of lncRNAs.

More importantly, other lncRNAs have been pointed as regulators of monocyte differentiation into the macrophage lineage, thus likely acting as suppressors of osteoclastogenesis. A recent study by Yang et al.^114^ demonstrated that the lncRNA NTT favors monocyte differentiation into the macrophage lineage by controlling the expression of the PBOV-1 gene. Authors found that PBOV-1 overexpression led to an increase in the number of adherent human THP-1 cells, which suggests their differentiation into macrophages, increasing as well the percentage of cells expressing CD68, a classical macrophage marker. In addition, NTT knockdown was accompanied by a decrease in PBOV-1 expression, which was due to the incapability of NTT-driven binding of hnRNPU to the PBOV-1 gene promoter^114^. However, these observations should be considered carefully, since the phenotypic features observed in the differentiated macrophages may also be shared by osteoclasts, requiring the verification for the absence of markers and functions more specific of these cells. In an earlier study, HOTAIRM1 was demonstrated to be a myeloid lineage-specific lncRNA and to increase upon retinoic-acid-induced differentiation of human myelocytic cells into the granulocytic lineage^115^. The next step in evaluating the role of these lncRNAs in osteoclastogenesis would be to monitor their expression upon differentiation of the same myeloid precursors into the osteoclast or the macrophage lineages. Moreover, further transcriptome-wide comparative studies of osteoclasts and macrophages differentiated from the same myeloid precursor are still needed in order to uncover the whole lncRNA network that regulates and determines osteoclastogenesis in detriment of macrophage differentiation.

### LncRNA regulation of other biological processes linked to osteoporosis

Besides focusing solely on lncRNAs involved in osteoblastogenesis and osteoclastogenesis, novel therapies for osteoporosis may be focused on other pathways involved in disease etiology. For instance, vitamin D is an important regulator of bone homeostasis^116^, and recent studies suggest it might not only affect the expression of lncRNAs, but its action may also be affected by lncRNAs. Jiang et al.^117^ showed that vitamin D receptor deletion, and thus interference with vitamin D signaling pathways, changes the transcriptional profile of several lncRNAs in mouse keratinocytes. Similarly, Riege et al.^118^ reported an alteration in lncRNA expression in human monocytes challenged with different pathogens, upon stimulation with vitamin D. On the other hand, the lncRNA H19 was described to inhibit vitamin D receptor in colon cancer, in a mechanism dependent on miR-675-5p, conferring resistance to vitamin D treatment^119^.

Moreover, the therapeutic potential of lncRNAs in osteoporosis goes beyond the resolution of the primary mechanisms underlying the disease, extending to secondary conditions arising from the osteoporotic phenotype. In fact, bone fragility in osteoporosis patients is a major cause of aggravated bone fragility fractures^120^. Interestingly, in the work of Huang et al.^121^, sequencing of RNA from femur subchondral tissues revealed a different gene-expression pattern between patients suffering from femoral head osteonecrosis and patients with femoral neck fracture, including for 602 lncRNAs. Although alterations in lncRNA expression in fragility fractures compared to standard fractures still need to be explored, this finding suggests that bone fracture might be associated with a specific lncRNA signature, which may constitute new therapeutic targets.

lncRNAs have also been described to participate in several biological processes that take place after bone fracture, and that are crucial for proper bone healing, namely inflammation and angiogenesis^122^. In the last decades, different lncRNAs were shown to promote or suppress inflammatory responses, but in the context of this article, only those affecting inflammatory pathways implicated in bone homeostasis are explored^123^. Although the role of lncRNAs in the resolution of fragility fractures still remains undetailed, several of these molecules have been detected altered in bone inflammatory conditions, as osteoarthritis and rheumatoid arthritis, representing potential targets for inflammation modulation in injuries occurred in osteoporotic patients. HOTAIR, H19, and linc-p21 are examples of such lncRNAs^123,124^. HOTAIR was found down-regulated in synoviocytes of rheumatoid arthritis patients^125^. Its overexpression in rat chondrocytes decreased the secretion of IL-17 and IL-23, diminishing also the percentage of T_h_17 pro-inflammatory cells upon in vivo injection in a rat model of rheumatoid arthritis, and reducing the levels of phospho-p65, IL-1β, and TNF-α in cartilage from the same animals^126^. linc-p21 is also decreased in blood samples of rheumatoid arthritis patients, but an increase in its expression in human T cells, as induced by methotrexate, reduced NF-kB activation^127^. Importantly, several other lncRNAs, such as NKILA, HOTAIR, ANRIL, linc-p21, NEAT1, among others, are described to control NF-kB signaling, a key pathway in inflammatory events^128^, contributing to the resolution of inflammation, a process necessary for proper bone healing.

The relationship between lncRNAs and inflammatory processes that affect bone has been further evidenced in tissue samples from osteoarthritis patients. In the work of Pearson et al.^129^, the expression of the lncRNAs PACER, CLinc01 and CLinc02 in hip and knee cartilage of osteoarthritis patients was decreased compared with healthy controls. Moreover, the stimulation of a chondrocyte cell line knocked-down for CLinc01 or CLinc02, with the pro-inflammatory cytokine IL-1β significantly increased the secretion of pro-inflammatory cytokines, relative to control-transfected chondrocytes under the same pro-inflammatory conditions^129^, revealing a regulatory role of these lncRNAs in cytokine secretion. In addition, Wang et al.^130^ reported that the lncRNA POIR, a pro-osteogenic lncRNA, was downregulated in periodontal MSC of patients affected by periodontitis, a bone disease characterized by a chronic pro-inflammatory environment, and that this expression alteration was a consequence of inflammation. In fact, inflammation was associated with a high expression of miR-182, a negative regulator of POIR^130^. The transition of macrophages from the M1 to the M2 phenotype during bone fracture healing is also an important step of inflammation resolution, and crucial for the success of bone repair. Although the participation of lncRNAs in regulating this process in vivo remains to be demonstrated, in vitro work revealed a promising role for these RNAs to ameliorate bone fracture repair via inflammation modulation. In fact, human primary macrophage polarization in vitro into the M1 pro-inflammatory phenotype, or the M2 pro-regenerative phenotype was shown to be accompanied by changes in the expression of lncRNAs, for instance TCONS_00019715 and THRIL. More interestingly, knockdown of TCONS_00019715 was confirmed to promote the transition of M1 THP-1-derived macrophages into the M2 phenotype^131^. In addition, Atianand et al.^132^ demonstrated that lincRNA-EPS is able to repress the expression of pro-inflammatory genes in murine bone-marrow-derived macrophages, which suggests this lncRNA might also play a role in the resolution of inflammation that establishes upon bone fracture. Finally, lncRNAs were also shown to control fibroblast inflammation in a context of tissue injury, namely in cornea^133^. In fact, downregulation of NEAT1 suppressed the secretion of pro-inflammatory cytokines, such as TNF-α and IL-6^133^, two key cytokines during bone fracture healing, suggesting that the knockdown of this lncRNA in fibroblasts present in the provisional matrix deposited in fracture sites following injury, might also contribute to inflammation resolution and proper bone regeneration.

On the other hand, few lncRNAs are described as direct or indirect regulators of endothelial cells activity, promoting angiogenesis. In fact, the lncRNAs LINC00323 and MIR503HG were previously shown to be upregulated in HUVECs conditioned in an hypoxic environment. In addition, their knockdown inhibited the capacity of endothelial cells to form capillary structures in vitro^134^. Similar observations were also reported for MALAT1^135^. Interestingly, MALAT1 is also capable of regulating the angiogenic regulatory capacity of MSC, with MALAT1 overexpression in MSC contributing to an increase in VEGF secretion and, consequently, enhanced capacity of conditioned media from these cells to promote in vitro angiogenesis of HUVECs^136^.

From the literature, it is evident that the role of lncRNAs in the control of processes such as inflammation and angiogenesis are still elusive in the context of fragility fractures occurring in osteoporotic patients. However, from the studies here described in the context of other bone disorders, it becomes clear that novel therapeutic strategies for osteoporosis using lncRNAs to simultaneously modulate processes involved in bone homeostasis will likely have synergistic effects, promoting an improved therapeutical outcome.

### lncRNAs in animal models of osteoporosis

Several animal models of osteoporosis have been used to mimic the mechanisms of the disease in vivo, including estrogen deficiency-induced osteoporosis, glucocorticoid-induced osteoporosis, and disuse osteoporosis^137^. To study postmenopausal osteoporosis, ovariectomy (OVX) that causes estrogen deficiency is the basis for the most commonly used animal model^137^. Although dozens of studies focused on understanding miRNA deregulation following OVX in rat and mice, very few have analyzed lncRNA expression levels. In fact, the work of Hao et al.^138^ was one of the first studies performing an integrative analysis of RNAs expression in OVX animals, including of lncRNAs. The expression of mRNA, miRNA, and lncRNA was profiled in the mandible of OVX mice, with a set of lncRNAs being positively correlated with miRNA-targeted genes, and another set being negatively correlated, potentially acting as ceRNAs^138^. Among these, mmu_1281_PI428960544 and mmu_18087_PI428960544 were identified as potential regulators of risk genes of osteoporosis development^138^. More recently, analysis of OVX-derived MSC versus sham-derived MSC revealed a significant up-regulation of the lncRNA brain-derived neurotrophic factor antisense (BDNF-AS) in the OVX group during osteogenic differentiation induction^139^. This transcript is antisense of BDNF coding gene, a neurotrophin most known for its key role in central  and peripheral nervous system development and maintenance, but that has also been reported as a promoter of bone formation and healing^140^. Feng et al.^139^ further elucidated the role of BDNF-AS in osteogenesis, showing that BDNF expression is up-regulated, while its lncRNA antisense transcript (BDNF-AS) is gradually downregulated, during 14 days of osteogenic differentiation induction of mice MSC in vitro. The reverse correlation of these transcripts was further confirmed by functional assays whereby BDNF-AS upregulation decreased BDNF at mRNA and protein levels. Moreover, BDNF-AS overexpression inhibited MSC osteogenic differentiation, but induced proliferation of undifferentiated cells^139^.

Also, using OVX as a model, Wang et al.^141^ identified MEG3 as overexpressed in BM-derived MSC from OVX mice compared with sham-operated mice. This finding has also been validated in MSC isolated from postmenopausal women with osteoporosis compared with premenopause healthy women^141^. MEG3 levels positively correlate with miR-133a-3p, which expression is decreased during the early stages of MSC osteogenic differentiation^141^. MEG3 overexpression is able to restore miR-133a-3p levels to the same levels found in undifferentiated cells and decrease the levels of its target gene (SLC39A1), suggesting silencing of MEG3 or miR-133a-3p could be a strategy to promote bone formation^141^. However, this is not in agreement with the previous studies reporting a pro-osteogenic role for MEG3^83,85^. In another study using OVX mice, authors suggest DEP domain containing mTOR interacting protein (DEPTOR) to negatively regulate MEG3, and confirmed MEG3 as a promoter of osteogenesis, by upregulating the BMP4 signaling pathway^142^.

In addition, Wang et al.^143^ recently described a role for the lncRNA LINC00311 in the activity of osteoclasts in OVX rats. The intraperitoneal injection of LINC0031-expressing vector in OVX mice significantly decreased the BMD of lumbar vertebrae, femur and tibia, comparing with sham-operated animals, which was accompanied by an increase in the number of TRAP-positive cells in bone tissue^143^. In accordance with these findings, in vitro overexpression of the lncRNA LINC00311 through transfection of osteoclasts differentiated from bone-marrow cells of the OVX rats, decreased cell apoptosis, increased proliferation, and increased the number of active TRAP-positive cells in vitro, in comparison with mock-transfected and nontransfected cells^143^. At the molecular level, the effect observed in OVX rat-derived osteoclasts upon transfection of LINC00311-overexperessing vector was accompanied by a decrease in DLL3, NOTCH1, Jagged and Hes-1, but an increase in NOTCH2 and TRAP, at the mRNA and protein levels^143^. Similarly, the expression of these genes was also found to be altered in bone tissues of OVX-LINC00311 rats compared with OVX-control rats^143^.

Although used to a much lesser extent than the OVX osteoporosis models, a few studies have also demonstrated the deregulation of lncRNAs in other models of the disease, namely disuse osteoporosis. In a rat model of hindlimb unloading, the expression of H19 was shown to be markedly downregulated in the affected limb, in comparison with control animals. This effect was likely mediated by Wnt signaling pathway inactivation, prompted by the up-regulation of Dkk4^144^. More recently, the same research group showed that this H19 downregulation may be caused by its hypermethylation^144^, although the causal relationship between bone mechanical loading and DNMT1 upregulation is still not clearly dissected. Of note, to the best of our knowledge, a lncRNA deregulation in glucocorticoid-induced osteoporosis models has not been reported thus far.

### LncRNAs as circulating biomarkers in osteoporosis

**Fig. 3:**
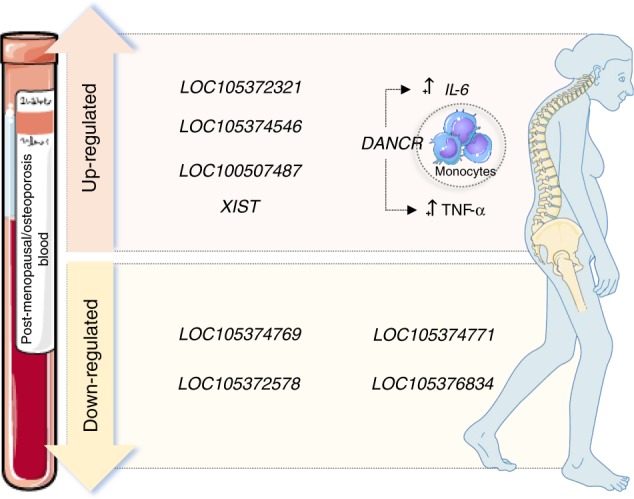
Differently expressed long noncoding RNAs in blood samples are potential biomarkers for osteoporosis

LncRNAs have been explored in different areas for their potential as biomarkers of disease diagnosis and prognosis^145–152^, particularly in the cancer field^153^. Surprisingly, few studies have addressed the value of lncRNAs as biomarkers in osteoporosis in humans, and only recently a correlation between the two was suggested, with studies focusing their analysis on blood samples (Fig. 3)^154–156^.

A recent study by Chen et al.^72^ reported the levels of the lncRNA XIST are higher in peripheral blood monocytes from osteoporosis patients than from normal subjects. However, these findings should be carefully interpreted and further confirmed, since the clinical features of patients and the control group are not clearly defined, and sample processing before XIST levels analysis is not extensively documented.

Previously, a more robust study by Fei et al.^154^ profiled the expression of lncRNAs by RNA sequencing in blood samples of postmenopausal women diagnosed with osteoporosis and found that 51 transcripts were significantly deregulated relative to samples from healthy women. From these, LOC105372321, LOC105374546, and LOC100507487 were the most upregulated lncRNAs, whereas LOC105374769, LOC105372578, and LOC105374771 were the most downregulated. Moreover, the simultaneous analysis of the mRNAs differently expressed between both groups revealed that the expression of several of these mRNAs was highly correlated with lncRNA expression^154^. Importantly, Gene Ontology enrichment and KEGG pathway bioinformatics analysis correlated the mRNAs differently expressed in postmenopausal women to biological processes such as inflammatory response, osteoclast differentiation, and cytokine–cytokine receptor interaction, among others. Moreover, some of these mRNAs were located within a distance of 100-kb to lncRNAs, including ALP, that was located nearby LOC105376834, with both transcripts being downregulated in postmenopausal women^154^. Together, these findings suggest a *cis*-regulation of the expression of bone metabolism related mRNAs by lncRNAs. Nonetheless, these results should be carefully considered, since only three women with postmenopausal osteoporosis and two healthy women controls were included in the study^154^. Still, this work is one of the first profiling whole blood lncRNAs associated with osteoporosis in humans, paving the way for further exploiting blood lncRNAs as biomarkers for the diagnosis of osteoporosis, and monitoring of disease progression under different treatment regimens.

In line with these results, Tong et al.^155^ had previously shown that osteoporosis was related with changes in the expression of specific lncRNAs in peripheral blood monocytes of postmenopausal women. In this work, it was found that the lncRNA DANCR is overexpressed in monocytes isolated from postmenopausal women with low BMD compared with women with high BMD^155^. Considering circulating monocytes can differentiate into osteoclasts^157,158^, the correlation of DANCR levels with BMD feature suggests DANCR as a potential biomarker in osteoporosis^155^. In addition, its overexpression in monocytes promoted an increase in IL-6 and TNF-α mRNA and secreted protein levels, whereas knockdown of DANCR in monocytes isolated from low-BMD women caused the opposite effect on those cytokines^155^. Of note, both cytokines are implicated in osteoporosis pathology, with TNF-α promoting RANKL-induced osteoclast formation^159^ and IL-6 stimulating osteoclastogenesis^160^. Furthermore, IL-6 and TNF-α levels were also correlated with DANCR expression in low-BMD osteoporosis patients^155^. Importantly, cell culture media from monocytes overexpressing DANCR increased bone-resorbing activity in mouse bone cultures, which could be neutralized by anti-IL-6 or anti-TNF-α treatments^155^. The mechanism underlying DANCR-IL-6/TNF-α link should be further dissected, and explored for new osteoporosis treatments.

The analysis of lncRNAs in plasma/serum for the diagnosis/prognosis of osteoporosis has been much more challenging. So far, only the study by Chen et al.^72^ presented above reported XIST as being upregulated also in the serum of osteoporosis patients. Although plasma/serum is as readily accessible as whole blood, the quantity of lncRNAs circulating in this biofluid in different pathological conditions has been suggested to be very low^161,162^, which might compromise their analysis by easily implementable and affordable techniques, delaying their establishment in the clinics as cell-free circulating biomarkers for osteoporosis diagnosis and prognosis.

### LncRNA-associated SNPs and risk of osteoporosis

**Fig. 4:**
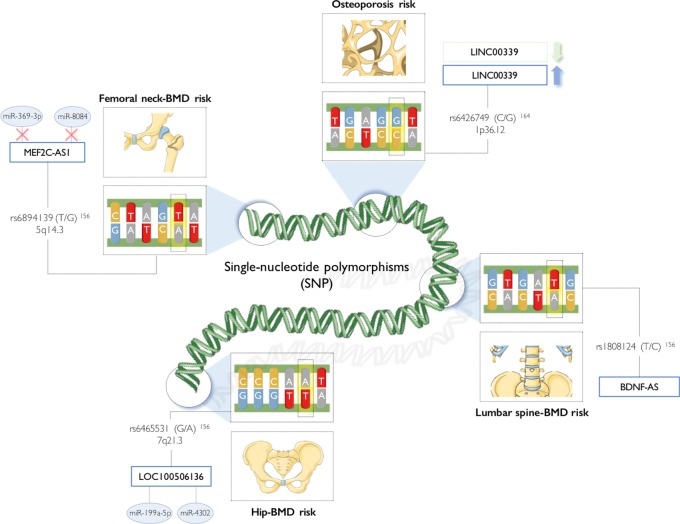
Single-nucleotide polymorphisms in long noncoding RNAs are associated with bone mineral density and osteoporosis risk

Interestingly, associations of lncRNAs with osteoporosis were also suggested to occur at the DNA level, with SNPs in coding and noncoding genes being identified as determinants of BMD, and thus as potential biomarkers of risk of osteoporosis development, accessible by a simple genetic test^156,163^. The most relevant lncRNA SNPs associated to osteoporosis and fracture risk are detailed in Fig. 4.

In early studies, SNPs in the genomic region 1p36 was found to be inversely correlated with hip and spine BMD, and positively associated with low-trauma osteoporotic fracture^163^. At the time, no known gene was mapped to this region. Most recently, Chen et al.^164^ further explored the genetic variants of this region and validated the association of rs6426749 (C/G) SNP at 1p36.12 with lower BMD, proposing it as a major risk factor for osteoporosis. Interestingly, the authors reported this region acts as an enhancer that regulates in *cis* the expression of the lncRNA LINC00339, which in turn inhibits the expression of CDC42^164^, a player in bone metabolism^165^. Recently, a meta-analysis of large-scale genome-wide association studies also identified 26 specific loci corresponding to lncRNAs that are potentially associated with BMD, and thus osteoporosis. From these, Zeng et al.^156^ found a significant association of the SNP rs6894139 (T/G) in the lncRNA MEF2C antisense RNA 1 (MEF2C-AS1) with femoral neck BMD, and of the SNP rs6465531 (G/A) in the lncRNA LOC100506136 with total hip BMD. Interestingly, simulations of lncRNA secondary structure predicted that rs6894139 SNP on MEF2C-AS1 may disrupt the binding site of miR-369-3p and miR-8084, whereas rs6465531 SNP on LOC100506136 may originate binding sites for miR-4302 and miR-199a-5p (pro-osteogenic miRNA)^156^. In addition, the SNP rs1808124 (T/C) in BDNF-AS was also found to be significantly associated with lower lumbar spine BMD^156^ in the GEFOS (Genetic Factors for Osteoporosis Consortium^166^).

### LncRNA gene therapy strategies and their therapeutic impact in osteoporosis

Considering the roles of lncRNAs in controlling bone metabolism, it is tempting to explore them as target regulatory molecules in the development of novel therapies aiming to treat osteoporosis. However, the translation of lncRNAs into the clinics is still in its infancy, including in the field of osteoporosis and other musculoskeletal disorders. According to clinical trials official registries (www.clincialtrials.gov), only 25 clinical trials are registered that evaluate the role of lncRNAs in disease^167^, being most of them dedicated to establish lncRNAs as biomarkers for diagnosis and prognosis, and not as therapeutic molecules. Furthermore, these trials encompass mainly cancer and cardiovascular patients, with none evaluating patients with musculoskeletal disorders.

The therapeutic application of lncRNAs has been precluded by the limited knowledge on their biological function that only in recent years has been further clarified, and also by constraints common to gene therapies. Among these, the low efficiency of in vivo transgene transfection, the recurrent use of immunogenic gene delivery vehicles, and the unpredictable and uncontrollable behavior the transgene might have in vivo, often leading to malignancies, are among the major hurdles, still to be overcome for widespread clinical application of gene therapy^168^. These motivate the development of innovative and more effective strategies to interfere with lncRNA expression in vivo, which will likely be applicable to other disorders, including osteoporosis.

Considering the biological roles of lncRNAs, therapies may aim to promote their expression and/or action, or to inhibit it. For lncRNA overexpression, constructs containing the lncRNA of interest or its regulatory sequences (Table 1) are delivered by either viral or nonviral strategies, for cell transfection in vivo. Sidi et al.^169^ reported the treatment of bladder cancer patients by overexpression of the H19 promoter, and a toxin under its regulation. The BC-819 plasmid used consisted of a double-stranded DNA construct carrying the H19 promoter sequence and the diphtheria toxin A DNA, and was intended to be expressed in cancer cells, which usually express H19 at high levels. To improve transfection upon delivery, the plasmid was complexed with polyethyleneimine and then instilled into patients' bladder. In this study, several mild to moderate adverse effects were observed, and importantly, 44% of the patients had complete marker tumor ablation, supporting the transcription effectiveness of the plasmid^169^. In another study, Chen et al.^170^ used the *sleeping beauty* transposon-based baculovirus hybrid system for the expression of the lncRNA PTENP1 in mice. This system is composed by one baculovirus vector containing the PTENP1 transgene, and another baculovirus vector containing the transposase responsible for the incorporation of the transgene into the host genome. The intratumoral injection of this system in an orthotopic mouse model of hepatocellular cancer had a tumor suppressive effect, promoting cell apoptosis and inhibiting cell proliferation^170^. Chang et al.^171^ also reported an alternative system for MEG3 lncRNA overexpression, based on MS2 bacteriophage virus-like particles (VLPs) crosslinked with the GE11 polypeptide, which binds the EGFR receptor, facilitating particles internalization and thus cell transfection. These VLPs carrying the MEG3 gene were successfully administered in a mouse model of hepatocellular cancer, leading to tumor growth inhibition in vivo^171^. On the other hand, lncRNA expression may be inhibited by different antisense methods, with many in vitro studies reporting the use of small interfering RNA (siRNA), shRNA, and antisense oligonucleotides (ASOs), most of which can be directly injected for in vivo delivery, or associated with a delivery system (usually nonviral)^172^. The expression of MALAT1 was reported to be knocked-down in vivo using siRNA complexed with the commercially available liposome-based vehicle *invivofectamine****®***, which was injected in the vicinity of an orthotopic tumor in a mouse model of chemoresistant prostate cancer, inhibiting the growth of chemoresistant tumors^173^. Most recently, Hu et al.^174^ described a novel approach in the lncRNA field, whereby functionalized single-wall carbon nanotubes were used for the delivery of anti-MALAT1 ASOs, in a mice model of multiple myeloma. ASOs-loaded nanotubes were injected intratumorally or intravenously in two different models of the disease, at different timepoints after tumorigenesis induction, resulting in tumor cell apoptosis and decreased tumor burden^174^. In another recent study, downregulation of the lncRNA KCNQ1OT1 was achieved by injection of lentiviruses carrying shRNA, demonstrating the role of this lncRNA in the establishment of cardiotoxicity in mice, caused by the chemotherapeutic drug arsenic trioxide^175^. Taking into account the several methodologies available for lncRNA knockdown, the choice for a specific strategy is usually determined by the location of the lncRNA to be targeted, the efficiency and specificity of each form of synthetic nucleic acid used and the duration intended for their action (Table 1). Upon delivery to target cells, the double-stranded siRNAs are bound by Ago2 and incorporated into the RNA-induced silencing complex (RISC). Here, they are disassembled into single-stranded RNAs that bind the target lncRNAs by base complementarity, in particular those transcripts located in cell cytoplasm^176,177^, promoting their cleavage^178^. However, siRNA delivery to target cells has to be extensively optimized, since naked siRNAs are cell-impermeable, unstable in circulation and highly susceptible to degradation in vivo by serum nucleases, being also reported to activate pro-inflammatory responses (reviewed by Liu et al.^178^). On the other hand, ASOs are single-stranded oligonucleotides, binding target lncRNAs in various locations in the cell by base complementarity, majorly inducing their degradation by RNase H1. Due to their structure, these oligonucleotides are more easily internalized by target cells, which also favors their use to target nuclear lncRNAs (reviewed by Crooke et al.^179^). More importantly, they are considered to be more specific than siRNAs^179^. Unlike siRNAs and ASOs, shRNAs are delivered as double-stranded DNA constructs contained in plasmids, which are then transcribed and processed inside target cells much like pri-miRNAs, into small RNA molecules with a organized secondary structure. These small RNAs are then loaded into the protein complex RISC and promote lncRNA degradation in a mechanism similar to siRNAs (reviewed by Moore et al.^180^). The major advantage of shRNAs resides in the fact that, unlike siRNAs and ASOs, they can be transcribed along time, allowing a longer-term therapeutical effect. Moreover, due to their mode of action and organized secondary structure, shRNAs were previously suggested to have less off-target genes than siRNAs^181^.

**Table 1:** Possible strategies for modulation of long noncoding RNAs expression

Transgene technology	Advantages	Limitations	References
*lncRNA overexpression*			
Double-stranded DNA Plasmid	• Compatible with viral and nonviral vectors • Tested in vitro and in vivo	• Double-stranded DNA constructs only • Construct size limits choice of delivery vector and transfection efficiency	Chang et al.^171^ Chen et al.^170^ Sidi et al.^169^
EV-based	• RNA/DNA constructs restricted to the lncRNA sequence • Compatible with double-stranded DNA constructs • Does not need any additional delivery vehicle, but may be combined with biomaterials • Some degree of cell targeting	• Delivery of additional molecules besides lncRNAs, without a defined composition	Ma et al.^182^ Silva et al.^122^ Teixeira et al.^183^
CRISPR based	• Permanent genomic alterations • Tested in vitro and in vivo	• Incompatible with transient and timely controlled gene therapies • High risk of affecting secondary genes • Delivered as double-stranded DNA plasmids	Liu et al.^192^
*lncRNA underexpression*			
siRNA	• Compatible with viral and nonviral vectors • Tested in vitro and in vivo • May be chemically modified to improve pharmacokinetics and pharmacodynamics	• Less effective targeting nuclear lncRNAs • Highly susceptible to degradation if not conjugated with a delivery vehicle • Short-term effects only	Lennox et al.^176^ Liu et al.^178^ Wang et al.^173^
shRNA	• Suitable for longer-term effects • Can be expressed in the cell nucleus • Tested in vitro and in vivo	• Delivered as double-stranded DNA plasmids • Usually requires a viral vector for highly effective delivery	Jiang et al.^175^ Moore et al.^180^ Rao et al.^181^
ASO	• Compatible with viral and non-viral vectors • Tested in vitro and in vivo • More effective in targeting nuclear lncRNAs • More effective targeting nascent transcripts of lncRNAs • May be chemically modified to improve pharmacokinetics and pharmacodynamics	• Short-term effects only • Some degree of off-targets still observed	Crooke et al.^179^ Hu et al.^174^ Lennox et al.^176^ Vickers et al.^177^
CRISPR-based	• Permanent genomic alterations • Allow a bidirectional and *in cis* control of lncRNA gene expression • Tested in vitro and in vivo	• Incompatible with transient and timely controlled gene therapies • Lower specificity • High risk of affecting secondary genes	Baliou et al.^193^ Chen et al.^164^ Goyal et al.^195^ Liu et al.^192^

**Fig. 5:**
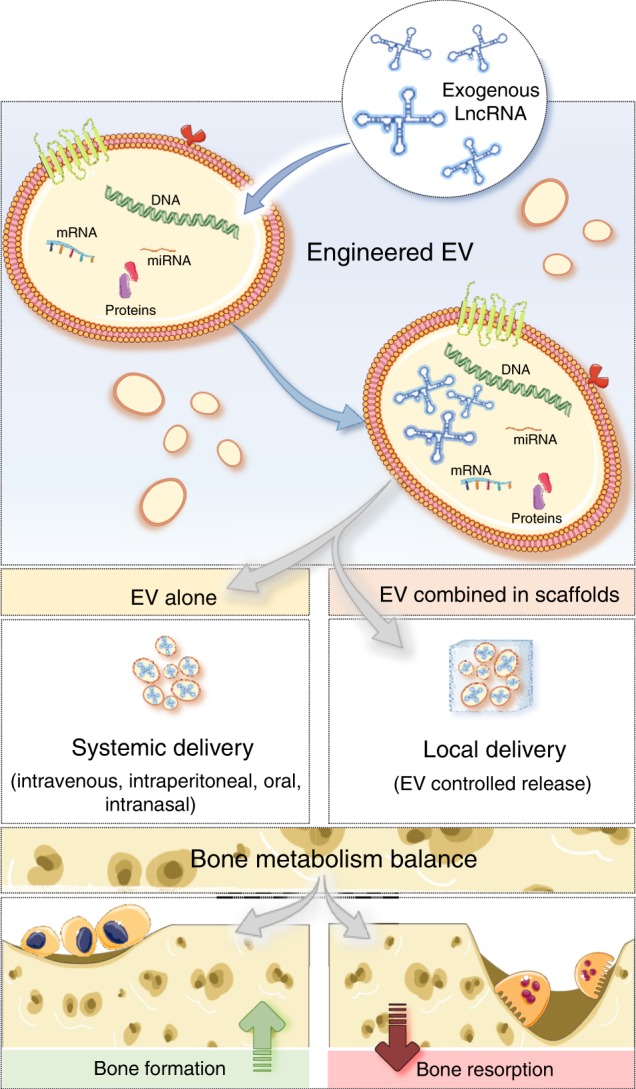
A potential delivery strategy for long noncoding RNA (lncRNA) is proposed. Extracellular vesicles (EV) are naturally secreted by cells and contain proteins, DNA, and RNA. Exogenous lncRNA capable to promote bone formation and inhibit bone resorption can be encapsulated into EV, which can be used as natural delivery vehicles. In vivo delivery of lncRNA-loaded EV can be systemic through intravenous, intraperitoneal, oral or nasal routes, or local through EV encapsulation into scaffolds, which might help to promote local bone repair upon fragility fractures

Overall, studies available suggest that lncRNA overexpression is usually more technically challenging and controversial than their downregulation, with the later benefiting from advances in the siRNA and miRNA fields. In fact, lncRNA overexpression usually requires vectors and delivery systems able to carry longer transgenes and with higher efficiency of transfection, comparing to the oligonucleotides used for lncRNA knockdown. An alternative to circumvent the drawbacks of cell transfection in vivo is the transfection of target cells in vitro, which are then transplanted for therapy. In fact, many of the in vivo studies published exploring the biological role of lncRNAs follow this approach. More importantly, lncRNAs have been found in extracellular vesicles (EVs) released by cells, constituting a natural method of lncRNA delivery into cells of interest^182^ (Table 1). Furthermore, EVs are suggested to have a certain degree of targeting, being preferentially internalized by specific cell types depending on their cell of origin^122^. In addition, they can be engineered to contain specific molecules of interest, including RNAs and drugs^183^. Therefore, EVs have been investigated as tissue-targeted delivery vehicles. Moreover, several works have been describing the capacity of MSC and osteoclasts to internalize EVs of different origin and capable of modulating osteogenesis^184–188^ and osteoclastogenesis^187,188^, suggesting EVs may function as vehicles for lncRNAs of interest involved in the regulation of bone metabolism (Fig. 5).

Another way to overcome the technical limitations impairing lncRNA overexpression or downregulation in vivo is related with the lncRNAs capacity to specifically recruit/bind proteins, such as PCR2^189^ and PUMILIO^190^, which suggests their activity may also be regulated by compounds able to bind the target lncRNAs in a similar way. Therefore, much like to the miRNA field, an investment in research aiming to find further drugs capable of targeting lncRNAs, such as small molecules or structurally homolog decoy proteins, should be further pursued^191^. Interestingly, the natural capability of lncRNAs to interact with proteins and other ligands open the possibility they may also be explored as carrier-like moieties for drugs and proteins of interest, including compounds for osteoporosis treatment, that could be administered systemically.

In more recent years, the advances in genome editing recurring to CRISPR/Cas9 technology have also opened new doors for the regulation of lncRNAs expression in human cells, more permanently, at the gene level^192^. In fact, CRISPR/Cas9 extends the possibilities of lncRNA expression modulation initiated by the strategies that target mainly RNA (Table 1). The first tests in human patients to evaluate the safety and effectiveness of such approach are just starting, with the first clinical trial registered under USA approval only in August 2018, and focusing on the modification of the erythroid lineage-specific enhancer of the BCL11A gene of autologous CD34^+^ cells, infused back to β-thalassemia patients (www.clinicaltrials.gov). So far, lncRNAs gene editing via CRISPR/Cas9 has been most explored in vitro, with Chen et al.^164^ using this approach to demonstrate the enhancer activity of the genomic region rs6426749, implicated in the development of osteoporosis, over the lncRNA LINC00339, as described above. Furthermore, several studies have been using CRISPR/Cas9 techniques to modulate lncRNA expression in vivo, namely in different rodent models of human diseases^193^. Although CRISPR/Cas9 was not explored until now in models of osteoporosis neither of bone development and metabolism regulation, it was previously employed to study lncRNAs implicated in cell differentiation and tissue formation^194^, suggesting their applicability also in bone and bone-related diseases. Nonetheless, it should be noted that the complex architecture of lncRNAs, with transcripts overlapping coding genes with key functions in cell biology, might favor the use of RNAi-based therapeutical approaches targeting lncRNAs, instead of CRISPR-mediated gene editing, since they represent a more specific approach with reduced risks of deregulating neighbor genes^195^.

### Future perspectives

Although there are no reports on the use of lncRNAs in osteoporosis therapies up to date, the regulatory role these molecules have in the different types of cells that maintain bone homeostasis and participate in bone healing, turn them into promising molecular targets and therapeutical molecules to diagnose and treat osteoporosis. In the near future, lncRNAs may become particularly important as biomarkers for the detection of osteoporosis in human patients, since data has been consistently showing their potential as diagnostic/prognosis tools, particularly in the cancer field^196–198^, but also in rheumatic diseases^124^ that affect bone. Importantly, osteoporosis diagnosis/prognosis based on the detection of lncRNAs levels by standard techniques, may constitute a precise, accurate and objective method of diagnosis and staging of the disease that, together with the routine radiographic-based methods currently applied, may help to improve clinical decisions. Therefore, clinical trials engaging high number of patients should be carried out so that specific lncRNAs for osteoporosis diagnosis and prognosis could be uncovered. This approach could also be helpful for the validation of SNPs impacting osteoporosis risk. Furthermore, with the use of next-generation sequencing (RNA-seq) in clinical samples, the number of lncRNAs involved in osteoporosis, including the detection of novel transcripts, is expected to rapidly increase. In parallel, it is expectable that the use of lncRNAs as therapeutic tools in in vivo models of disease^199^ will be further explored. On the other hand, the translation of these research findings into human clinical trials will likely take longer time. Considering the fast advances in the field of gene therapy through CRISPR-based genome editing, it is possible that the use of lncRNAs as therapeutic tools for osteoporosis may also arise by manipulations at the genome level, in parallel with gene expression modulation by different RNAi-based strategies.

## Conclusion

Knowledge on biology, function and potential of lncRNAs as biomarkers and treatment targets in osteoporosis is still in its infancy. More studies raging from the basic biological mechanisms-of-action, to methods for their improved detection and in vivo therapeutic delivery are paramount. However, the results reported so far and the technological advances on this research field are promising for the treatment of osteoporosis.
